# Linguistic validation of translation of the self-assessment goal achievement (saga) questionnaire from English

**DOI:** 10.1186/1477-7525-10-40

**Published:** 2012-04-23

**Authors:** Elisabeth Piault, Sameepa Doshi, Barbara A Brandt, Çolpan Angün, Christopher J Evans, Agneta Bergqvist, Jeffrey Trocio

**Affiliations:** 1 MAPI Values, 133 Portland St, Boston, MA 02114, USA; 2 Pfizer Inc, 235 East 42nd Street, New York, NY 10017, USA; 3 Corporate Translations, Inc, East Hartford, CT 06108, USA; 4 Pfizer PIO, VetenskapsvΣgen 10, SE-19190, Sollentuna, Sweden; 5 Pfizer, Inc, 235 East 42nd Street 235/9/42, New York, NY 10017, USA

**Keywords:** SAGA, Validation, Lower urinary tract symptoms, Overactive bladder, Goal achievement, Patient-reported questionnaire

## Abstract

**Background:**

A linguistic validation of the Self-Assessment Goal Achievement (SAGA) questionnaire was conducted for 12 European languages, documenting that each translation adequately captures the concepts of the original English-language version of the questionnaire and is readily understood by subjects in the target population.

**Methods:**

Native-speaking residents of the target countries who reported urinary problems/lower urinary tract problems were asked to review a translation of the SAGA questionnaire, which was harmonized among 12 languages: Danish, Dutch, English (UK), Finnish, French, German, Greek, Icelandic, Italian, Norwegian, Spanish, and Swedish. During a cognitive debriefing interview, participants were asked to identify any words that were difficult to understand and explain in their own words the meaning of each sentence in the questionnaire. The qualitative analysis was conducted by local linguistic validation teams (original translators, back translator, project manager, interviewer, and survey research expert).

**Results:**

Translations of the SAGA questionnaire from English to 12 European languages were well understood by the participants with an overall comprehension rate across language of 98.9%. In addition, the translations retained the original meaning of the SAGA items and instructions. Comprehension difficulties were identified, and after review by the translation team, minor changes were made to 7 of the 12 translations to improve clarity and comprehension.

**Conclusions:**

Conceptual, semantic, and cultural equivalence of each translation of the SAGA questionnaire was achieved thus confirming linguistic validation.

## Background

Overactive bladder (OAB) is a chronic condition with a prevalence of approximately 11% in men and 13% in women in Europe [1]. Despite a significant armamentarium of effective agents available for the treatment of overactive bladder, many patients with OAB symptoms do not discuss their symptoms with their healthcare provider and remain untreated. In addition, once symptoms are identified and patients are treated, they often discontinue treatment when their expectations are not achieved [2]. Successful management of OAB and lower urinary tract symptoms (LUTS) may be contingent on patients having realistic treatment goals and on clinicians understanding which treatment goals are most important, for example, those symptoms that have the greatest severity or debilitating impact on patients' lives.

The Self-Assessment Goal Achievement (SAGA) questionnaire is a communication tool designed to help patients with LUTS and their healthcare providers adopt reasonable treatment expectations and assess goal achievement [3-5]. Developing realistic treatment expectations and attaining those goals may lead to improved treatment outcomes, a concept observed in other therapeutic areas [6,7]. Briefly, the baseline SAGA module asks patients to rate the importance of 9 prespecified (fixed) treatment goals that describe reducing the following LUTS: frequency (daytime and nighttime); sensation of pressure; primary sensation to use the bathroom; bladder voiding; starting or maintaining a urine stream; urine loss when coughing, laughing, exercising, or sneezing; urine leakage; and urgency. In addition to the 9 fixed goals, patients can list up to 5 additional (open) treatment goals. Patients rate the importance of each goal using a 5-point scale ranging from "not very important goal" to "very important goal." In the SAGA follow-up module, patients rate their achievement of each individualized goal and overall goal achievement with a 5-point scale ranging from "did not achieve goal" to "greatly exceeded goal." SAGA is the first questionnaire to capture individualized treatment expectations and goals regarding LUTS [8,9].

European regulatory bodies have raised concerns over the validity of measures developed in one language and then used in other languages [10]. The European Regulatory Issues and Quality of Life Assessment (ERIQA) Group recommends that a rigorous approach is taken in the translation of patient-reported outcome (PRO) measures for use in international settings to achieve conceptual and semantic equivalence across languages [11]. Guidance for gaining this evidence is provided in a publication generated by the International Society for Pharmacoeconomics and Outcomes Research (ISPOR), entitled *Multinational Trials - Recommendations on the Translations Required, Approaches to Using the Same Language in Different Countries, and the Approaches to Support Pooling the Data: The ISPOR Patient-Reported Outcomes Translation and Linguistic Validation Good Research Practices Task Force Report*,[12] which was developed to expand on a previous publication (2005) that provided guidance for the translation of PRO measures [13]. Because there exists an increased need to translate and culturally adapt PRO measures, the document details areas designed to maintain content integrity during translation. Similarly, the US Food and Drug Administration recommends that language translations of PRO instruments being used in countries where the language is not the same as the country in which the instrument was developed show evidence that the content validity and other measurement properties of the instrument remain equivalent to those of the original version [14].

A harmonized translation of the SAGA questionnaire was created in 12 languages: Danish, Dutch, English (UK), Finnish, French, German, Greek, Icelandic, Italian, Norwegian, Spanish, and Swedish. The objective of the present study was to conduct a linguistic validation of the SAGA questionnaire for those 12 European languages to document that each translation adequately captures the concepts of the original English-language version of the questionnaire and is readily understood by subjects in the target population.

## Methods

### Participants

Participants were recruited by clinical investigators through referrals. Participants had to be native-speaking residents of the target countries who reported urinary problems/lower urinary tract problems. Subject selection criteria for each language, assuming a sample of 8 participants per language, were patients with self-reported urinary problems/lower urinary tract problems, equal representation of gender, and (when possible) ≥ 2 participants with fewer than 12 years of education, ≤ 2 participants per 10-year age range and a minimum range of 30 years between the youngest and oldest subject, and ≥ 3 geographical areas in the target country.

### Linguistic validation

**Figure 1:**
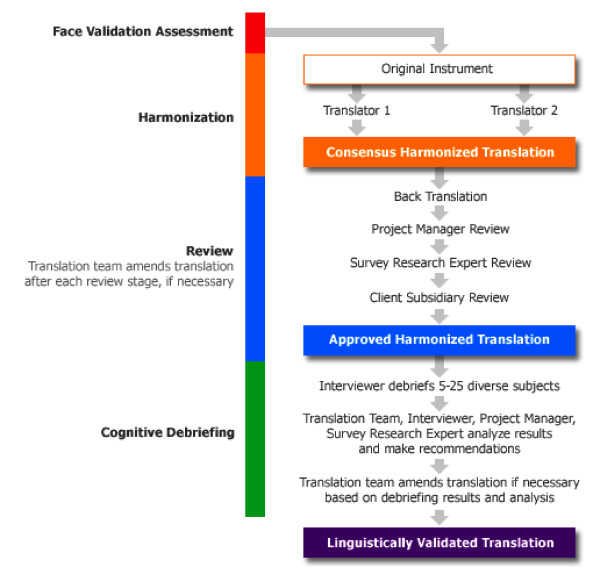
**Overview of a typical linguistic evaluation process**.

A schematic overview of a typical linguistic validation process is illustrated in Figure 1. The methodology to conduct the linguistic validation of SAGA was based on linguistic adaptation principles detailed by the European Regulatory Issues and Quality of Life Assessment (ERIQA) Group [11] and the International Society for Pharmacoeconomics and Outcomes Research (ISPOR) [12,13] and recommended by the US Food and Drug Administration [14].

Translations were created by linguists meeting the cultural, technical, and linguistic standards of competence through a process of forward and back translations and review by an expert in survey research and a local user, in accordance with industry standards outlined in Appendix A. A harmonized translation of the SAGA questionnaire was created in 12 languages: Danish, Dutch, English (UK), Finnish, French, German, Greek, Icelandic, Italian, Norwegian, Spanish, and Swedish. The translations were then tested via cognitive debriefing interviews of patients with OAB residing in the target countries. Cognitive debriefing is a standardized interview conducted by a trained interviewer following a subject's review and completion of a PRO instrument. All interactions with participants were conducted in the target language only. The source language was never used in discussing the document with the subject. Bilingual interviewers (English and target language) were trained to conduct one-on-one cognitive debriefing interviews as previously described [15].

### Study procedures

Participants were asked to review the SAGA questionnaire during a baseline interview and in the follow-up module, to return the questionnaire with any words, phrases, or sentences circled that were difficult to understand. During a subsequent debriefing telephone interview, participants were asked to paraphrase each sentence in the questionnaire and explain why any circled words were difficult to understand. Comprehension rates (defined as total percentage of subjects who were able to successfully paraphrase the items within this instrument) were captured during debriefing interviews. Interviewing was conducted using a script that was read to the participants: "As you know, we are testing a questionnaire for use in clinical studies and want to know if they can be easily understood. Did you have a chance to review the document and circle any words that you found difficult to understand or any questions that were difficult to answer?" The interviewer judged whether items were correctly paraphrased and recorded any comprehension problems or proposed changes to the wording. In keeping with regulatory guidelines and good clinical practice, cognitive debriefing information was captured on a data collection form (Appendix B).

In the subsequent qualitative analysis, linguistic validation teams, consisting of the original translators, back translator, project manager, interviewer, and survey research expert, evaluated the debriefing results. The teams categorized problems that emerged during the debriefing as: Conceptual - a function of the original English; Linguistic - a function of the words used to translate the English concept; or Stylistic - a function of the subject's preference for a different wording. When warranted, the original translators of the questionnaire created a new harmonized translation of problem words or sentences and the back translator created a new back translation for review by a survey research expert. Once all issues were resolved, final forward and back translations were created.

## Results

### Participants

After creating comprehensive translations, which were approved by the translators, project manager, and survey research expert involved in its production, debriefing interviews were conducted with 144 participants with urinary/lower urinary tract problems from 16 countries: Denmark, The Netherlands, Finland, United Kingdom, Ireland, Greece, Norway, Iceland, Spain, Sweden, France, Belgium, Luxembourg, Switzerland, Italy, Germany. The participants ranged in age from 18 to 87 years and had 4 to 24 years of education (Table 1). An equal number of men and women participants in each language sample were included. The age range in the Danish population was < 30 years (60-79 y).

**Table 1:** Demographic characteristics of the participants by target language*

Language	Residence (number of towns/cities)	Age (years)	Gender (males/females)	Education (years)
Danish	Denmark (8)	60 - 79	4:4	7 - 15

Dutch	Belgium, Netherlands (16)	31 - 85	8:8	6 - 16

English	Ireland, United Kingdom (10)	29 - 71	8:8	11 - 18

Finnish	Finland (4)	21 - 77	4:4	13 - 24

French	Belgium, France, Luxembourg, Switzerland (24)	25 - 86	16:16	5 - 19

German	Germany, Switzerland (15)	27 - 78	8:8	9 - 18

Greek	Greece (3)	32 - 87	4:4	4 - 16

Icelandic	Iceland (6)	23 - 70	4:4	14 - 18

Italian	Italy (5)	40 - 74	4:4	5 - 17

Norwegian	Norway (6)	20 - 76	4:4	7 - 16

Spanish	Spain (6)	18 - 55	4:4	9 - 18

Swedish	Sweden (7)	27 - 73	4:4	10 - 16

* n = 8 for each country

### Cognitive debriefing

A breakdown of item comprehension by language is summarized in Table 2. The average item comprehension rate for each item in the SAGA questionnaire, across languages, was 98.9%. A summary of the comprehension difficulties for each SAGA item is listed in Table 3. For most difficulties, the terms were judged by the translation team to not require changes because the overall comprehension levels were relatively high and the translated terms were accurately rendered in the target languages. The various types of changes that were required are summarized in Table 4 with the majority of changes made to the questionnaire instructions. No changes were made for the Danish, Spanish, and Swedish translated versions. For the Greek, Italian, Norwegian, German, and English (Irish and UK) versions, comprehension difficulties that required changes occurred only in the instructions for the questionnaire. For example, "Finally, circle the three most important goals in the combined symptom and other personal goals sections, as shown below" was the most common statement that was incorrectly paraphrased, with an 88.1% comprehension rate. Therefore, this instruction was changed for greater conceptual clarity. Examples of other changes include: for the Dutch translation, all changes were made to the instructions, including a comma inserted after "treatment goals" for greater conceptual clarity: "Each patient is unique and has different treatment goals, depending on the symptoms experienced, lifestyle, and any other health concerns" and "Use the scale below..." was replaced by more common terms for the target countries. This revision was made throughout the questionnaire. In the French version, examples of changes made were: in the instructions, "Circle the 3 goals from the combined sections below (Symptom and Other personal goals) which are the most important for you" was revised for greater conceptual clarity; and in the item "To reach my goals I will:" the phrase "Keep record of my progress" was revised as "Keep track of my progress" for both greater conceptual clarity and cultural appropriateness. The Icelandic version had 2 changes to improve grammatical accuracy: in the item "Reduce the difficulty starting or maintaining a urinary stream" a preposition was added, and in the item "Sign your name to confirm your commitment to working towards your own better health" noun case was changed.

**Table 2:** Item Comprehension Rate by Language

Language	n	Comprehension Rate, %
Danish	8	100

Dutch	16	97.8

English	16	100

Finnish	8	99.9

French	32	98.8

German	16	99.8

Greek	8	99.7

Icelandic	8	99.3

Italian	8	94.1

Norwegian	8	96.8

Spanish	8	100

Swedish	8	100

**Overall**	144	98.9

**Table 3:** Comprehension Difficulties by SAGA Item

Item	Category	Incorrectly Paraphrased	ItCR
INITIAL ASSESSMENT	Title	3	97.9%

Each patient is unique and has different treatment goals depending on the symptoms experienced, lifestyle, and any other health concerns.	Instructions	3	97.9%

A goal is something that you would like to improve by treatment.	Instructions	1	99.3%

It may be a symptom that you would like to improve, or an activity that you cannot do because of your urinary symptoms.	Instructions	1	99.3%

This questionnaire, the Self-Assessment Goal Achievement Questionnaire, is designed to record your goals for the treatment of your urinary problems.	Instructions	10	93.0%

There are two sections to complete.	Instructions	3	97.9%

The first, symptom goals, relates to the typical urinary symptoms of patients like you; you may have all or some of these symptoms.	Instructions	2	98.6%

The second section, other personal goals, relates to the specific impact on your life from your urinary symptoms.	Instructions	2	98.6%

Examples of other personal goals include being able to visit family and friends for a longer period of time before having to find a restroom, or to reduce the number of times it is necessary to change clothes due to urine loss.	Instructions	1	99.3%

Please use the questionnaire to indicate which goals are important to you and how important each goal is by marking the appropriate box beside each goal.	Instructions	3	97.9%

Finally, circle the three most important goals in the combined symptom and other personal goals sections, as shown below.	Instructions	17	88.1%

Take the completed form with you when you see your healthcare provider so that together you can discuss your urinary problems, your treatment goals, review your treatment options, and develop and commit to your treatment plan.	Instructions	1	99.3%

Your treatment goals - First Visit	Title	1	99.3%

Use the scale below which ranges from 1 (not important goal) to 5 (very important goal).	Instructions	1	99.3%

Circle the three goals from the combined sections below (Symptom and Other personal goals) which are the most important for you.	Question	17	88.1%

Reduce my urine loss when I have a sudden need to rush to the bathroom	Response Option	1	99.3%

Other personal goals	Response Option	1	99.3%

(not included above and important to me)	Response Option	3	97.9%

Once you and your healthcare provider have discussed your goal(s) and developed a plan of action, mark the actions below which will apply to you.	Question	7	95.1%

Sign your name to confirm your commitment to working towards your own better health.	Instructions	1	99.3%

Read health tips about my condition	Response Option	2	98.6%

Keep record of my progress	Response Option	5	96.5%

FOLLOW-UP ASSESSMENT	Title	11	92.3%

The initial assessment provided you with an opportunity to discuss your treatment goals related to your urinary problems with your healthcare provider.	Instructions	1	99.3%

Since that discussion, have you reached your goals?	Question	1	99.3%

First, look over the treatment goals you and your healthcare provider discussed during your last visit.	Instructions	1	99.3%

Much worse than expected	Response Option	2	98.6%

As expected	Response Option	2	98.6%

Much better than expected	Response Option	2	98.6%

After you have completed the questionnaire, take it with you when you see your healthcare provider so that together you can discuss your treatment goal achievement and further management of your urinary symptoms.	Instructions	2	98.6%

Your treatment goal achievement - Follow-up Visit	Title	1	98.6%

CUT OUT THE GRAY SQUARE to see your other personal goals	Administrative	12	99.3%

When answering the following question, please think about all of your goals.	Question	1	97.6%

Overall, to what extent have you achieved your goals?	Question	1	99.3%

ItCR = item comprehension rate.Note: No changes were made for Spanish, Danish, and Swedish languages.

**Table 4:** Types of Changes Required To Resolve Comprehension Difficulties

Language	Spelling/Grammar	Comprehension	Conceptual	Instructions	Cultural Appropriateness
Dutch	X	X	X	X	

English	X			X	X

Finnish					X

French			X	X	X

German	X		X	X	

Greek			X	X	

Icelandic	X				

Italian			X	X	

Norwegian		X	X	X	X

## Discussion

The primary validation study of the SAGA questionnaire has recently demonstrated that the instrument helped patients adopt reasonable treatment expectations and assess goal achievement [16]. Telephone interviews, which were used as a means of obtaining information in this study, have been shown to be acceptable means of capturing accurate information in other medical disciplines [17,18]. Multiple language iterations of the SAGA questionnaire should facilitate wide-spread utilization of this PRO instrument in subjects with LUTS. The results of the cognitive debriefing interviews indicated that the translations of the SAGA questionnaire into 12 European languages adequately captured the concepts of the original English-language version of the questionnaire and were readily understood by participants with urinary/lower urinary tract problems.

Recently, various PRO measures for assessing OAB were translated and assessed for linguistic validity by McKown et al.,[19] including the Nocturia Quality of Life (N-QOL) questionnaire, Overactive Bladder Questionnaire (OAB-q) family, Patient Perception of Bladder Condition (PPBC) questionnaire, Overactive Bladder Satisfaction Questionnaire (OAB-S) and International Consultation on Incontinence Questionnaire (ICIQ) Male Sexual Matters associated with Lower Urinary Tract Symptoms Questionnaire (ICIQ-MLUTSsex). Like the findings reported here for the SAGA questionnaire, McKown et al. found that the translations were well-understood by in-country, native-speaking subjects [19]. Also consistent with our findings for the SAGA questionnaire, a number of minor changes were made to the N-QOL, OAB-q, OAB-S, and ICIQ-MLUTSsex translations to improve clarity and cultural appropriateness.

The SAGA questionnaire was developed based on goal attainment scaling methods to facilitate the patient-provider interaction and the tailoring of a treatment plan based on individual patients' goals, with the aim of increasing patient satisfaction and improving therapeutic outcomes [20]. The SAGA questionnaire provides a patient perspective on treatment goals and expectations that is not attained with other instruments assessing LUTS. The concepts of "treatment goals and expectations" are novel for PRO measures, and different cultures use varying terms to capture this information. Both the identification and prioritization of treatment goals and the translation and understanding of the language nuances are critical for successful gathering of this information. For example, while the term "goal" is used in the United States, the term "objective" is used in Europe, so the latter term is used, where appropriate, in the translated questionnaires for cultural appropriateness and greater conceptual clarity. In the instructions for the questionnaires, various language iterations had the word "goals" replaced.

The SAGA questionnaire is expected to improve healthcare provider-patient communication and treatment outcomes in clinical practice and be useful for assessing goal achievement in clinical trials. Multiple language iterations should help communication between patients and physicians in different countries, which could lead to more consistent assessment and treatment of the LUTS that are most bothersome to individual patients. Time spent to ensure that patients are able to understand each question being asked, especially considering the variations among languages, is expected to help guide physicians with treatment decisions. The measurement properties of the translated versions will be presented at upcoming international research symposia.

## Conclusions

Translations of the SAGA questionnaire from English to 12 European languages adequately captured the concepts in the original English version of the questionnaire, thereby demonstrating the conceptual, semantic, and cultural equivalence of each translation. Participants with LUTS demonstrated an ability to understand the concepts in the questionnaire, with an overall comprehension rate of 98.9%. Based on the results of cognitive debriefing interviews of participants reporting LUTS, minor changes were made to 7 of the 12 translations to improve clarity and comprehension.

## Competing interests

The authors declare that they have no competing interests.

## Authors' contributions

EP and CJE were involved in the tool design; BAB and ÇA were involved in assessment and interpretation of translated versions; EP, SD, BAB, ÇA, CJE, AB and JT contributed to intellectual content development, writing, and critical review of the manuscript.

## Appendix A. Appendix A. Translation and Harmonization Procedures (V1.0)

1. Two translators who are native speakers of the target language and are experienced in translating health questionnaires independently translate the document.

2. After both translations are complete, the two translators compare their translations and produce a third translation jointly. This process of discussion and review is known as "harmonizing" and the resulting third translation is referred to as the "harmonized translation."

3. The harmonized translation is then given to a native English-speaking translator for translation back into English. This document is referred to as the "back-translation."

4. A project manager compares the original English to the back-translation and either approves or questions each item in the back-translation.

5. The project manager discusses concerns with the three translators, who may change the translation, change the back-translation, or leave the translation as it is, providing a justification to the project manager. A new harmonized translation and back-translation may be created.

6. A survey research consultant (or document author, if available) compares the original English to the harmonized back-translation and either approves or questions each item.

7. If portions of the back-translation are not approved, then the translation is sent back to the three translators who may change the translation, change the back-translation, or leave the translation as it is, providing a justification to the survey research consultant (or author). This process is repeated until all translation issues have been resolved and the revised back-translation is approved.

8. Local users selected by the client review the translation to identify any additional concerns. If the local user requests changes, the steps are repeated until all concerns have been addressed and all revisions are approved by the survey research consultant (author).

## Appendix B. Appendix B. Sample Data Collection Form (DCF)

The sample DCFs illustrate the two steps in the collection and analysis of cognitive debriefing data, as follows:

1. Interviewers complete and submit a DCF for each subject as specified in the *Cognitive Debriefing Manual*.

2. Interviewers compile DCFs into a Summary DCF and submit the Summary DCF.

The interviewer should label the Summary DCF with the language code, e.g., SW-SW Summary DCF is the label for the Swedish for Sweden Summary DCF.

At the top of the DCF, the interviewer enters the interview begin and end times. The subject's demographics and the time to complete the interview (elapsed time) are entered into the boxes that correspond to the subject's ID#. On the Summary DCF, the interviewer enters the demographic information for all participants.

**Table:** Interview Begin Time: ___:____ Interview End Time ___:____

Subject	1	2	3	4	5
**Age (years)**					

**Gender**					

**Academic Education (years)**					

**City and country of residence**					

**Elapsed time for interview**					

**Item** **1**	**Circled Items** **2**	**Paraphrased** **Correctly/Concept Understood** **3**		**Comments** **4**	

**Modified Brief Pain Inventory-Short Form (mBPI-sf)**		YES			

1. Have you experienced any pain in the past 24 hours?		YES		Two subjects preferred the wording "viimeksi kuluneiden 24 tunnin aikana" (during the **last **24 hours) and said it to sound "better Finnish". **Resolution: No change. Stylistic preference**.	

No		YES			

Yes		YES		Two subjects suggested changing the affirmative word "on" (yes) to the affirmative word "kyllä" (yes). Interviewer: "On" (yes) is more commonly used in spoken language, but it understandable as it is used here. **Resolution: No change. Stylistic preference**.	

If you answer NO to question one (1), please stop now.		YES		All subjects suggested changing both this item and the one following to past tense, since the question has already been answered. **Resolution: No change. Stylistic preference. Translation accurately reflects the original English and all subjects understood the concept**.	

If you answer YES, complete the questionnaire.		YES			

2. On this scale, how much pain are you having **right now**?		YES			

*No Pain*		YES			

*Worst pain possible*		YES			

3. On this scale, please indicate the **worst **pain you have had in the past 24 hours.		YES			

4. On this scale, please indicate the **average **level of pain you have had in the past 24 hours.		YES			

Please mark **X **the number below that describes how, during the past 24 hours, pain has interfered with your:		YES		Four subjects suggested changing "haittaa täysin" (completely interferes) to the present tense, or changing the responses to past tense, so the tenses of the question and responses are consistent. Interviewer agrees but translation reflects the source language so no change is recommended. **Resolution: No change. Stylistic preference. Translation reflects the original English and all subjects understood the concept**.	

A. General activity		YES			

Does not interfere		YES			

*Completely interferes*		YES			

B. Mood		YES			

C. Walking ability		YES			

D. Relations with other people		YES			

E. Sleep		YES			

F. Normal Work, including housework		YES		Two subjects suggested changing "taloustyöt" (housework) to "kotityöt" (homework) because it is more common and does not mean work done for school. Interviewer: "Taloustyöt" is not wrong but "kotityöt" is more common. **Resolution: Change accepted. Translation team agrees that "kotityöt" is a more common way of expressing the concept**.	

G. Enjoyment of life		YES			
